# Physical ability, cervical function, and walking plantar pressure in frail and pre-frail older adults: An attentional focus approach

**DOI:** 10.3389/fragi.2022.1063320

**Published:** 2022-12-08

**Authors:** Laurianne Pinloche, Qingshan Zhang, Sophie E. Berthouze, Karine Monteil, Christophe Hautier

**Affiliations:** ^1^ Université de Lyon, UCBL-Lyon 1, Laboratoire Interuniversitaire de Biologie de la Motricité, Villeurbanne, France; ^2^ Unité Recherche ISOstéo, Ecully, France; ^3^ School of Athletic Performance, Shanghai University of Sport, Shanghai, China

**Keywords:** functional capacity, aging, cervical, plantar pressure, TUG

## Abstract

Aging and increased vulnerability define the clinical condition of frailty. However, while the cervical function is recognized as a determinant of balance and walking performance, no study simultaneously physical ability, cervical function, balance, and plantar pressure distribution in walking in nursing house population. Thus, the present study aimed to compare these parameters between Frail and Pre-Frail aged people. Thirty-one (12 men and 19 women) institutionalized participants (age: 89.45 ± 5.27 years, weight: 61.54 ± 9.99 kg, height: 160.34 ± 7.93 cm) were recruited and divided into Pre-Frail and Frail groups according to SPPB (Short Physical Performance Battery) score (Frail <6, Pre–Frail ≥6). Participants performed the Timed Up and Go Test (TUGT) and a static balance evaluation. The cervical range of motion (COM), knee extensor strength, and walking plantar pressure distribution have been measured. The Pre-Frail group showed a higher gait speed (ES = 0.78, *p* ≤ 0.001) and a better TUGT, as well as higher knee extensor strength (ES = 0.4, *p* = 0.04). Furthermore, the Pre-Frail group presented a center of pressure (COP) displacement velocity on the sagittal axis (ES = 0.43, *p* = 0.02) and a more COP projection on this axis (ES = 0.43, *p* = 0.02). No significant difference has been observed between the two groups concerning the total contact time and most of the plantar pressure parameters except for the rear foot relative contact time which was lower in the Pre-Frail group. The Pre-Frail group also showed better cervical tilt mobility (ES = 0.35, *p* = 0.04). This study highlights the influence of some new parameters on frailty in older people, such as cervical mobility and plantar pressure distribution in walking.

## Introduction

Aging is a global phenomenon often occurring in altered living conditions, with loss of mobility leading to incapacity and primary care input. Increased vulnerability defines the clinical condition of frailty and concerns all psychological, physical, and social capacities (Gobbens et al., 2010). Deconditioning is intrinsically linked to balance disorders and loss of mobility threatening autonomy in daily life. Nursing home populations are particularly concerned by frailty, with people affected heterogeneously (Sverdrup et al., 2018). However, due to a high percentage of institutionalized elderly suffering from cognitive impairment, psychological and social determinants of frailty could be challenging to investigate.

Consequently, the physical aspects appear to be the most accessible parameters to evaluate if tests are adapted. A relationship has already been established between frailty and Short Physical Performance Battery (SPPB), even for complex elders living in nursing homes (Tabue-Teguo et al., 2018). There is the same kind of relationship between frailty and the well-known SPPB test as between SPPB and aging, with a decrease in walking pace, an increase in static imbalance (Xie et al., 2016), and a loss of muscular strength in the lower limbs (Barbat-Artigas et al., 2016). An age-related decrease in walking speed is associated with a high risk of falls (Barack et al., 2006). Elderly fallers also exhibit postural instability on both sagittal and transversal axes and modifications in the static center of pressure, displacement, and velocity (Muir et al., 2013).

Aging is also at the origin of modifications in a global posture with accentuation of forwarding inclination of the trunk, deeper kyphosis, general asymmetry, flexed knees, and ankles (Drzał-Grabiec et al., 2013). This phenomenon makes older adults cautious when walking, reducing speed, step length, and symmetry with increased step variability, frequency, and bilateral contact phase time Field (Iosa et al., 2014), which negatively correlated with gait performance. Few studies have explored plantar pressure distribution in older adults with or without imbalance issues but have identified higher peak pressures with age localized on the forefoot (McKay et al., 2017). More precisely, it seems that peak pressure at the heel pose and metatarsophalangeal joint at the toe-off decreases while the contact time of the same parts increases. These results are observed when comparing young and old participants (Scott et al., 2007) and fallers and non-fallers (Nakajima et al., 2014). However, no studies for now compared gait patterns according to the stage of frailty.

Since age impacts spine statics and postural control, modifications could lead to balance strategy maladjustment with weakened postural control, altered visual feedback, proprioceptive and vestibular system impairment, and neuromuscular trouble (Woollacott, 2000). Previous studies show that the neck area is an anatomical and physiological crossroads for the balance (Armstrong et al., 2008). The inclination capacity of the cervical spine appears decisive for adaptation to everyday movement, particularly in case of loss of equilibrium, and could become a marker of frailty. Moreover, the cervical function is the last possibility for the spine to adjust the balance with plantar proprioception decrease and to compensate for vestibular alteration. Spine mobility is commonly affected by aging, and it has been demonstrated that decreased cervical mobility and asymmetry in rotation can impact anteroposterior swing in standing position in older adults (Quek et al., 2013). Even in young participants, cervical muscle tiredness could alter static balance parameters by modifying the speed displacement of the center of pressure (COP) (Liang et al., 2014). In addition, over-activation of superficial neck muscles appears with aging and a global decrease of muscle tone to the detriment of deep muscles. This particular pattern leads to a forward position of the head (Gogola et al., 2014). It is well known that part of the trunk and neck role stabilizes the head and cushions the acceleration during walking (Kavanagh et al., 2006). The cervical area is affected by aging, structurally and functionally, therefore influencing static and dynamic balance. Nevertheless, to our knowledge, no balance rehabilitation program includes prevention, enhancement, or rehabilitation of this body part. Consequently, it appears very important to analyze the modifications in neck muscle strength and mobility regarding physical capabilities and balance in frail elderly participants.

While many studies have focused on different components of frailty in older adults, none have simultaneously analyzed, in nursing home populations, gait speed, validated mobility tests, lower limb strength, COP variations in static standing posture, dynamic distribution of plantar pressure, and cervical strength and mobility. Thus, the principal aim of the present study was to compare mobility and balance parameters between Frail and Pre-Frail (Frail vs Pre-Frail) groups in nursing homes to highlight specific differentiation criteria useful for individualized injury prevention or rehabilitation. It was hypothesized that older adults at different frailty levels would present different walking plantar pressure patterns and spine mobility associated with strength losses that could impair their physical ability.

## Materials and methods

### Experimental approach

This cross-sectional study was designed to compare the different physical abilities between Pre-Frail and Frail groups. Short Physical Performance Battery (SPPB) test, TUGT, knee extensor strength, cervical strength and range of motion, static balance, and walking plantar pressure were measured in the two groups. Each measurement was realized three times, and the best score was considered for analysis. Afterward, the participants were divided into Pre-Frail and Frail groups according to the SPPB threshold score of 6; thus, the Pre-Frail group (SPPB scores from 0 to 5) and the Frail group (SPPB scores from 6 to 12) (Pritchard et al., 2017).

### Participants

Thirty-one participants were recruited in three nursing homes, including 12 men and 19 women (age: 89.45 ± 5.27 years, weight: 61.54 ± 9.99 kg, height: 160.34 ± 7.93 cm). The inclusion criteria for the participant recruitment were over 65, able to walk 10 m, and understanding simple orders. They completed an information and consent form before participation in the study, approved by the Ethics Committee of Université Claude Bernard Lyon 1, and complied with the Declaration of Helsinki.

### Experimental sessions

#### Short Physical Performance Battery (SPPB) test

The Short Physical Performance Battery (SPPB) is an objective assessment tool for evaluating lower extremity functioning in older persons. The SPPB consists of three tests, including the ability to stand for 10 s with feet in 3 different positions (together side-by-side, semi-tandem, and tandem), the fastest gait speed, and the time to rise from a chair five times (de Fátima Ribeiro Silva et al., 2021).

#### Time up and go test (TUGT)

Subjects were required to rise from a chair, walk 3 m, turn around 180°, walk back to the chair, and sit down while rotating 180° (Barry et al., 2014). The time to perform the total test was measured and considered to assess the person’s mobility. During the trial, the person was expected to use any mobility aids they would typically require.

#### Strength of the knee extensor and cervical muscles

A handheld dynamometer (HHD) (MicroFET2, Hoggan, Salt Lake City, United States) was used to measure the maximal isometric force of the quadriceps muscle of the dominant limb and the maximal cervical force in the three axes. A « make test » was performed to obtain the maximal isometric force. For the knee extensor force measurement, the force was normalized by each subject’s body mass. The subject was also asked to perform cervical movements until maximal strength was reached (or 5 s) with resistance applied successively under the chin, under the occiput, at the right/left side of the mandible, and on the right/left side of the temporal. Four indicators were obtained: Flexion Strength, Extension Strength, Rotation Strength, and Tilt Strength.

#### Cervical mobility

Cervical mobility was obtained from a standard measuring tape (material information). The subject was placed in a neutral sitting position, looking straight ahead, back in contact with the chair back. Anatomic benchmarks were identified (tragus of ear, chin symphysis, anterior part of acromion, superior part of the sternum), and the subject was asked to perform cervical movements: flexion, extension, right/left rotation, and right/left tilt. Active range of motion (ROM) measurements were identified as the neutral distance difference between neutral and maximal movement expressed in centimeters (Chibnall et al., 1994) for 4 ROM measurements including cervical flexion, extension, rotation, and tilt.

#### Static balance measurement

The statics balance was measured using a posturographic platform (Fusyo, Medicapteur, Balma, France, 40 Hz) in the eyes open condition. The participants stood barefoot with two legs on the platform and were asked to look steadily at the fixed points on the wall in front of the participant. The center of pressure (COP) displacement was measured during 25.6 s (Bernard et al., 2010) and processed with the software W-IN POSTURO (Medicapteur, Balma, France). From COP displacement, several indicators were calculated, including the surface of displacement (SURF), the total length of displacement (LXY), length of displacement on the sagittal axis (LY), mean position on the transversal axis (X_mean_), mean position on sagittal axis (Y_mean_), length of displacement as a function of surface (LFS), the COP speed of displacement on the sagittal axis (VFY).

#### Foot pressure parameter measurement

**FIGURE 1:**
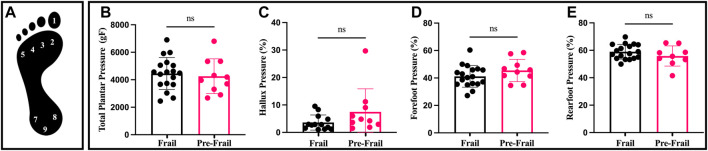
Plantar pressure and distribution; **(A)** sensors location on foot; **(B)** total plantar pressure; **(C)** percentage (%) of hallux pressure by total plantar pressure; **(D)** percentage (%) of forefoot pressure by total plantar pressure; **(E)** percentage (%) of rearfoot pressure by total plantar pressure. ns: non-significant.

Foot pressure was measured by W-INSHOE plantar sensors (Medicapteur, Balma, France, 100 Hz) during the walking phase of the Time Up and Go Test (TUGT) under standard conditions (Podsiadlo and Richardson, 1991). Nine pressure sensors were placed on the 3-foot locations, including hallux, forefoot, and rearfoot (Figure 1A). Data delivered by sensors concerned with pressure and duration. First, sensors were grouped according to their localization to compose the forefoot and the rearfoot to obtain a biodynamic pattern. Then, all parameters were normalized (%) according to total plantar pressure and total foot contact duration during the stance phase of the walking task. Six parameters were extracted: hallux pressure, forefoot pressure, rearfoot pressure, total pressure, rearfoot, and forefoot relative contact time. The average of the peak foot pressure of all the steps was calculated from the steps after stand-up for each location which was used for future analysis.

#### Statistical analysis

Prior to performing the statistical analysis, the Shapiro–Wilk and Levene’s tests were used to assess the data’s normality and variance equality for each variable. Non-parameter Wilcoxon test was used to determine the difference between the two groups (Pre-Frail vs Frail). The correlation coefficient r was calculated to estimate the effect size. The magnitude of the correlation coefficient was interpreted using criteria: very weak (0.11–0.19), weak (0.20–0.39), moderate (0.40–0.59), strong (0.60–0.79), and very strong (0.80–1.00). The critical *p*-value was set at 0.05. Descriptive statistics are presented as mean ± SD with 95% CI. All statistical procedures were performed with R software (R 3.5.0, R Core Team, Vienna, Austria).

## Results

**FIGURE 2:**
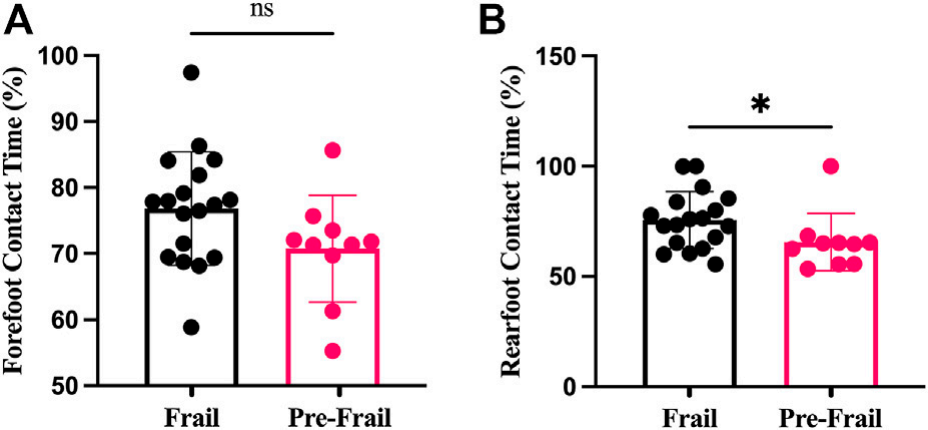
Plantar contact time; **(A)** forefoot contact time; **(B)** rearfoot contact time; ns: non-significant; *: *p* < 0.05.

The Pre-Frail group indicated significantly higher SPPB score, gait speed, and TUGT (all *p* < 0.002, ES > 0.74). In addition, the frail group revealed lower knee extensor strength compared to the Pre-Frail group (*p* = 0.05, ES = 0.4) (Table 1). Moreover, the Pre-Frail group showed a higher cervical tilt ROM (*p* = 0.04, ES = 0.4), whereas no significant differences were found in cervical flexion, extension, and rotation (all *p* > 0.05) (Table 1). In contrast, no significant difference was found for any cervical strength (all *p* > 0.05). The static balance results revealed that only VFY and Y_mean_ significantly differed between the two groups (*p* = 0.02, ES = 0.43). In contrast, no significant difference was found in other statics parameters (all *p* > 0.5). Lastly, Pre-frail group presented significant shorter forefoot contact time (*p* = 0.04, ES = 0.39, Figure 2), but no other significant difference was found for other parameters (all *p* > 0.05, Figure 1).

**TABLE 1:** Physical ability, cervical mobility, strength, static assessment, plantar pressure in Pre-Frail and Frail subjects. All data are presented as mean ± standard deviation with a 95% confidence interval (CI).

	Variable	Frail		Pre-frail		Difference [95% CI]	*p*-value	Effect size (r)	Magnitude
		Mean ± SD	[95% CI]	Mean ± SD	[95% CI]				
Physical ability	SPPB	3.2 ± 1.51	[2.49; 3.91]	7.18 ± 1.25	[6.34; 8.02]	3.98 [2.94; 5.03]	<0.001	0.82	Large
	Gait speed (m.s^−1^)	0.32 ± 0.08	[0.28; 0.36]	0.58 ± 0.13	[0.49; 0.67]	0.26 [0.17; 0.36]	<0.001	0.78	Large
	TUGT(s)	29.33 ± 16.8	[21.47; 37.19]	13.06 ± 3.74	[10.54; 15.57]	−16.27 [−24.4; −8.14]	<0.001	0.74	Large
	Knee extensor strength (N/kg)	1.66 ± 0.66	[1.31; 2.01]	2.33 ± 0.78	[1.68; 2.98]	0.67 [−0.02; 1.37]	0.05	0.4	Moderate
Cervical mobility	Flexion (cm)	7.55 ± 2.63	[6.32; 8.78]	8.27 ± 1.68	[7.14; 9.4]	0.72 [−0.86; 2.31]	0.43	0.15	Small
	Extension (cm)	4.75 ± 2.02	[3.8; 5.7]	4.91 ± 1.58	[3.85; 5.97]	0.16 [−1.19; 1.51]	0.69	0.08	Small
	Rotation (cm)	8 ± 2.15	[7; 9 ]	8.23 ± 1.94	[6.92; 9.53]	0.23 [−1.34; 1.79]	0.85	0.04	Small
	Tilt (cm)	3.38 ± 2.45	[2.23; 4.52]	4.91 ± 1.02	[4.22; 5.59]	1.53 [0.24; 2.82]	0.04	0.35	Moderate
Cervical Strength	Flexion Strength (N/kg)	1.26 ± 0.32	[1.04; 1.48]	1.26 ± 0.34	[1.01; 1.5]	0 [−0.31; 0.3]	1	0	Small
	Extension Strength (N/kg)	1.48 ± 0.27	[1.29; 1.66]	1.48 ± 0.5	[1.13; 1.84]	0.01 [−0.37; 0.39]	0.86	0.05	Small
	Rotation Strength (N/kg)	0.88 ± 0.33	[0.66; 1.1]	1 ± 0.36	[0.74; 1.26]	0.12 [−0.2; 0.44]	0.5	0.15	Small
	Tilt Strength (N/kg)	0.92 ± 0.27	[0.74; 1.1]	1 ± 0.32	[0.77; 1.23]	0.09 [−0.19; 0.36]	0.6	0.12	Small
Static assessment	SURF(mm^2^)	116.83 ± 91.15	[71.5; 162.15]	91.87 ± 58.61	[52.5; 131.25]	−24.96 [−82.05; 32.14]	0.74	0.07	Small
	LXY (mm)	534.03 ± 408.54	[330.86; 737.19]	319.66 ± 157.63	[213.76; 425.56]	−214.36 [−436.05; 7.33]	0.17	0.26	Small
	LY (mm)	264.11 ± 317.42	[106.26; 421.96]	185.45 ± 102.48	[116.6; 254.29]	−78.67 [−246.45; 89.12]	0.98	0.01	Small
	X_mean_ (mm)	58.17 ± 45.38	[35.6; 80.73]	35.54 ± 24.61	[19.01; 52.07]	−22.63 [−49.35; 4.09]	0.29	0.2	Small
	Y_mean_ (mm)	91.22 ± 43.56	[69.56; 112.89]	50.35 ± 44.81	[20.24; 80.46]	−40.87 [−76.19; −5.56]	0.02	0.43	Moderate
	LFS (mm)	1.19 ± 0.85	[0.77; 1.61]	0.74 ± 0.35	[0.51; 0.98]	−0.45 [−0.91; 0.02]	0.18	0.25	Small
	VFY(mm/s)	113.88 ± 64.2	[81.95; 145.8]	64.96 ± 45.72	[34.24; 95.67]	−48.92 [−90.98; −6.86]	0.02	0.43	Moderate
Plantar pressure	Hallux pressure (KgF)	3.6 ± 2.76	[1.93; 5.27]	7.5 ± 8.38	[1.51; 13.49]	3.9 [−2.21; 10]	0.08	0.38	Moderate
	Forefoot pressure (KgF)	2420.12 ± 666.41	[2088.72; 2751.52]	3183.56 ± 1673.62	[1897.1; 4470.01]	763.44 [−541.39; 2068.26]	0.14	0.29	Small
	Rearfoot pressure (KgF)	58.95 ± 5.33	[56.3; 61.61]	55.86 ± 7.37	[50.19; 61.53]	−3.1 [−9.09; 2.9]	0.4	0.17	Small
	Total pressure (KgF)	4453.62 ± 1161.2	[3876.17; 5031.07]	4255.41 ± 1266.68	[3349.28; 5161.53]	−198.22 [−1220.14; 823.71]	0.61	0.1	Small
	Forefoot contact time (%)	76.84 ± 8.61	[72.56; 81.12]	70.77 ± 8.07	[65; 76.55]	−6.07 [−12.88; 0.74]	0.11	0.31	Moderate
	Rearfoot contact time (%)	75.63 ± 12.9	[69.21; 82.04]	65.61 ± 13.11	[56.23; 74.99]	−10.01 [−20.79; 0.77]	0.04	0.39	Moderate

## Discussion

The present study aimed to determine whether there are differences in the measured parameters (mobility test, gait speed, knee extensor strength, static balance, plantar pressure, and cervical pressure) between Frail and Pre-Frail older adults. The main finding demonstrated that frail participants presented a significant difference in lower knee extensor strength and pressure distributions during walking, associated with an altered cervical function, especially in tilt mobility.

The results obtained in our nursing home population were in accordance with recent studies concerning TUGT or walking speed. Binotto et al. (2018) reviewed studies using gait speed as a marker of physical frailty in community elderly aged between 68 and 86 years. This review reported a systematic decrease in gait speed in frail people with a wide variability from 2.7% to 83.9%. In the same way, an association between functional test performance and knee extensor strength is well known, and a recent study by (Jacob et al., 2019) demonstrated the same repartition pattern according to SPPB score. Knee extensor force in the present study could not be compared to the literature due to our population specificity: older and more dependent than groups usually studied. However, the difference observed in maximal strength between Pre-Frail and Frail people associated with a lack of difference in BMI (body mass index) tends to indicate that dynapenia was more marked than sarcopenia in this population. Unfortunately, the present study did not enable the identification of the physiological determinants of this difference.

Static balance evaluation regarding frailty gave more heterogenous results. The Pre-Frail group had better static balance than the frail group: COP displacement velocity was a lower variable, and its projection on the anteroposterior axis was less retro-pulsed. This should enable lesser muscular stiffness and energy expenditure (Houdijk et al., 2009). Some previous results reported a similar finding, including the study by Wiśniowska-Szurlej *et al.* (Wiśniowska-Szurlej et al., 2019), which observed a negative correlation between frailty and LXY or VFY in 209 older adults. However, other studies like Marques *et al.* (Marques et al., 2019) reported no difference in sway or mean value of COP displacement between frail and pre-frail groups. These differences may be partly explained by the specific high-frailty status of nursing populations. . Consequently, frail people may be influenced by postural control alteration and daily activities, showing difficulties and increased risk of falls, especially when handling objects at a height or getting up from a chair (Barry et al., 2014).

To our best knowledge, while the gait pattern is well documented, to our best knowledge, a few study has compared plantar pressure distribution between frail and pre-frail individuals. For example, Scott *et al.* (Scott et al., 2007) compared the foot pressure and contact time during gait between 50 young and 50 older participants. They reported that older participants presented significantly decreased peak pressure on the heel, forefoot, and hallux and increased contact time on the heel and forefoot compared to younger people. Our results are in accordance with theirs concerning the tendency for a higher Hallux pressure in pre-frail participants and significantly higher rear foot contact time in frail subjects (*p* = 0.04). Even if pressure distribution and frailty have not been studied together, a comparison between elderly fallers and non-fallers can be made. Indeed, Nakajima *et al.* (Nakajima et al., 2014) also reported a reduction of plantar peak pressure in fallers and an extension of the double support phase. In the present study, the higher rear foot contact time measured in frail people suggested that this population presented similar patterns to elderly fallers with a shortening swing phase up to its elimination, leading to a shuffling gait. This is confirmed by the trend observed in hallux pressure, which tends to be higher in pre-frail people. More recently, Anzai et al., 2022 found that the classification of participants relative to their frailty state primarily relied on features obtained from the different plantar pressure during the walking in line with the present study (Anzai et al., 2022). Considering that it is commonly accepted that the forefoot and hallux are the propulsive part of the foot, it could be hypothesized that frail people no longer use them. Even if this result could be a consequence of plantar deformation, it was mostly due to a particular gait pattern. Various explanations could be given, such as plantar tissue stiffness, decreased strength, sensitivity or mobility of the foot, or alteration of the somatosensory system. Future studies are needed to explore these parameters. In consequence, it suggests that the measurement of the plantar pressures may be used as the new approach to evaluating aspects of the degree of frailty related to physical ability such as SPPB.

Whereas cervical function is the last possibility for the spine to adjust the changes induced by the foot postural entry, among others, to the best of our knowledge, it has never been reported in the literature in frail and pre-frail older people. Although Pan *et al.* (Pan et al., 2018) described a global decrease in all cervical mobility, discontinuous across age, they could not conclude reference values due to the large variability of results. Swinkels *et al.* (Swinkels and Swinkels-Meewisse, 2014) made the same conclusions and found that tilt and extension were not changed before 60 years. In the present study, only one parameter was significantly discriminant between frail and pre-frail: tilt mobility. Rotation or flexion mobility and strength would also be expected to be discriminant regarding previous findings (Quek et al., 2013; Liang et al., 2014), but this was not the case. Different hypotheses could be made: first, some authors explored the position of the head related to the trunk with passive stiffness. At the same time, we measured active mobility considering that it was more representative of daily life requirements. Moreover, as participants included in the present study were older and frailer than in the literature, more considerable variability in measurement could be hypothesized and make comparison difficult. Concerning cervical strength measurement, although HHD was painless and not intrusive, it did not allow differentiation of deep and superficial muscles, unlike an intra-muscular sensor, and this could explain the lack of differences observed in strength measurement.

Finally, tilt mobility seemed a relevant parameter because of its strong direct impact on the vestibule and inner ear orientation. Some neurophysiological hypotheses could be mentioned to explain the present results according to previous studies, which explored the influence of age on postural reflex. It is well known that aging provokes an alteration of vestibular structure, which could lead to so-called “vestibular omission” with a distortion of vestibulospinal and oculo-vestibular reflexes. In healthy participants, the cervical-ocular reflex increases to compensate for this loss (Kelders et al., 2003), and this reflex is mainly driven by rotation. The physiological compensation could be modified by diminished neck movement like hypokinesia, causing an increase in cervical-ocular and vestibular-ocular reflex (Ischebeck et al., 2018). Specifically, this capacity is less significant than the other movements (Watier, 2006), so it did not permit intra-movement compensation as rotation or extension did. Thus, the inclination capacity of the cervical spine is decisive for adaptation to everyday movement, particularly in case of loss of balance, and seems to be a marker of frailty.

## Limitation

The present study suffers some limitations, such as a relatively low number of participants, due to the inclusion and exclusion criteria, which excluded participants with cognitive impairment. However, this dimension is often altered in the elderly and drives to institutionalization, making recruiting a significant number of participants difficult. The further study requires recruiting more participants, especially younger participants, which permits confirmation of the current finding and investigates the impact of the age range on the current parameters. Finally, more features should be extracted from pressure data for a combined spatio-temporal analysis and have a deeper insight in gait quality alteration.

## Conclusion

In conclusion, as expected from the literature, some parameters like gait speed and muscular strength appear to be determinants for the level of frailty. Still, some new parameters, such as cervical tilt and plantar pressure distribution in walking, have also been observed. Considering that cervical mobility can be easily measured, it could become part of a clinical routine. Although plantar pressure measurements require specific equipment and competence, some professionals, such as podiatrists, could be involved in detecting frailty. Moreover, the combined use of technology and conventional support shows encouraging results in the prevention of falls (Giovannini et al., 2022). Further studies could enable exploration of the influence of cervical tilt and pressure plantar in walking on physical performance in older people. Evaluation or changes in one of these parameters should raise the attention of health practitioners and improve the individualization of prevention and rehabilitation programs.

## Data Availability

The original contributions presented in the study are included in the article/Supplementary Material, further inquiries can be directed to the corresponding author.
